# The physiological levels of epigallocatechin gallate (EGCG) enhance the Cd-induced oxidative stress and apoptosis in CHO-K1 cells

**DOI:** 10.1038/s41598-024-64478-7

**Published:** 2024-06-13

**Authors:** Ewa Wnuk, Iwona Zwolak, Elzbieta Kochanowicz

**Affiliations:** 1 Department of Biomedicine and Environmental Research, Institute of Biological Sciences, Faculty Medicine, The John Paul II Catholic University of Lublin, Konstantynów Ave. 1J, 20-708 Lublin, Poland; 2 Department of Molecular Biology, Institute of Biological Sciences, Faculty of Medicine, The John Paul II Catholic University of Lublin, Konstantynów Ave. 1I, 20-708 Lublin, Poland

**Keywords:** Cadmium, Epigallocatechin gallate, Cell viability, Mitochondrial membrane potential, Reactive oxygen species, Cell apoptosis, Biological techniques, Biotechnology, Cell biology

## Abstract

Currently, the increasing pollution of the environment by heavy metals is observed, caused both by natural factors and those related to human activity. They pose a significant threat to human health and life. It is therefore important to find an effective way of protecting organisms from their adverse effects. One potential product showing a protective effect is green tea. It has been shown that EGCG, which is found in large amounts in green tea, has strong antioxidant properties and can therefore protect cells from the adverse effects of heavy metals. Therefore, the aim of the study was to investigate the effect of EGCG on cells exposed to Cd. In the study, CHO-K1 cells (Chinese hamster ovary cell line) were treated for 24 h with Cd (5 and 10 µM) and EGCG (0.5 and 1 µM) together or separately. Cell viability, ATP content, total ROS activity, mitochondrial membrane potential and apoptosis potential were determined. The results showed that, in tested concentrations, EGCG enhanced the negative effect of Cd. Further analyses are needed to determine the exact mechanism of action of EGCG due to the small number of publications on the subject and the differences in the results obtained in the research.

## Introduction

**Figure 1:**
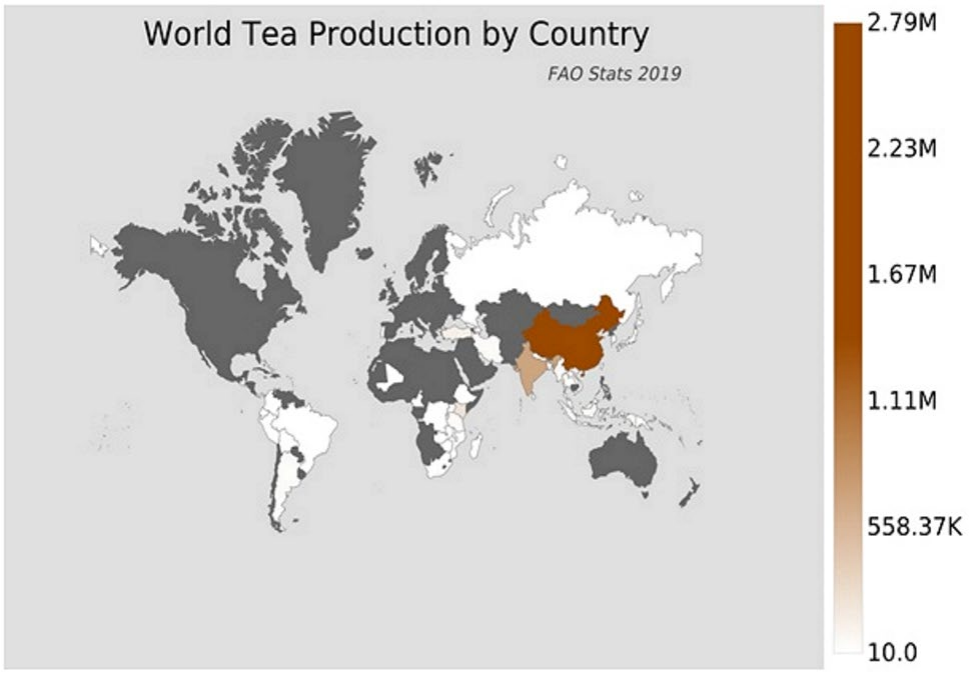
World tea production (https://www.atlasbig.com/en-ie/countries-by-tea-production).

Tea is one of the most popular beverages in the world. In 2019, tea production is estimated to be around 6.5 million tons per year. Asia accounts for 85.3% of tea production, with China being the largest producer both in Asia and globally (Fig. 1). There are many types of tea, such as green tea, black tea, white tea, oolong tea. Green tea extracted from two main plants in the *Theacease* family, *Camellia sinensis* and *Camellia assamica*^1^, is characterized by a wide range of medical benefits. Anticarcinogenic, antiapoptopic, antioxidant chelator of metals, and neuroprotective aspects are some of the widely reported benefits of this drink^2–5^. The beneficial effects of green tea on the organism have been linked to its compounds—catechins, which account for up to 35% of the dry weight of green tea leaves^1^. Eight major tea catechins are (+)-catechin (C), (−)-epicatechin (EC), (+)-gallocatechin (GC), (−)-epigallocatechin (EGC), (+)-catechin gallate (CG), (−)-epicatechin gallate (ECG), (+)-gallocatechin gallate (GCG) and (−)-epigallocatechin gallate (EGCG)^6,7^. Of these, EGCG is the most abundant compound. It accounts for approximately 70% of the total catechins in green tea^8,9^. It is over 3 times more than can be found in black tea^10^. It was estimated that in a cup of green tea (2.5 g green tea leaves in 200 mL of water) ± 90 mg of EGCG can be found^7^.

**Figure 2:**
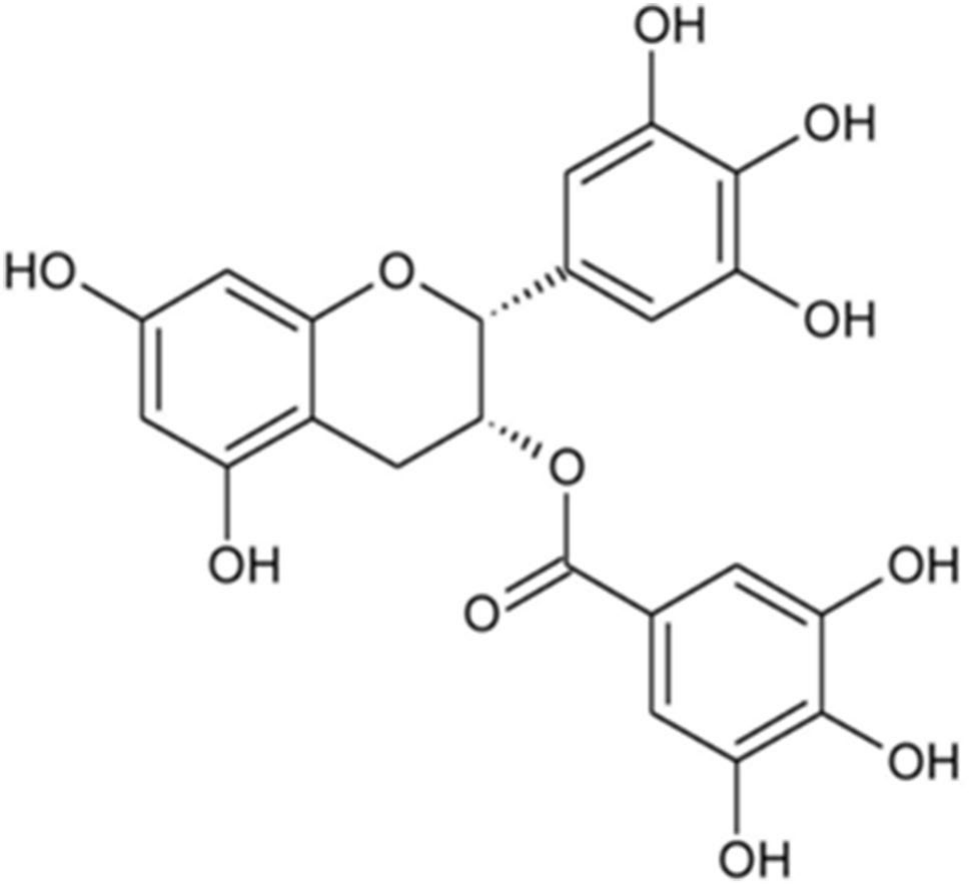
(−)-Epigallocatechin gallate (EGCG) structure.

The health benefits of EGCG, and therefore green tea, are mainly due to the structure of EGCG (Fig. 2). It has strong metal-chelating properties by having two active –OH rings that form stable metal complexes. Moreover, the phenolic and hydroxyl groups are responsible for scavenging ROS, resulting in antioxidant properties^11–13^. It was proved that the more –OH groups are in EGCG, more effective is the catechin’s free radicals scavenging^14^. Due to its nature, EGCG has been considered for treating heavy metal poisoning^15^. Based on studies on animals and cell culture models, it has been shown that EGCG possesses protective properties during exposure to heavy metals. A reduction in the amount of produced ROS, an increase in cell numbers and a reduction in the number of cells undergoing apoptosis were observed^16–20^. Despite the many reports of EGCG’s positive effects on cellular functions, there are also reports of harmful, toxic effects of this compound. Observations and analyses made it possible to rank the phenols from tea in order from the one showing the least cytotoxicity to the one with the highest: epicatechin < gallic acid, epigallocatechin < epicatechin-3-gallate < epigallocatechin-3-gallate^21^. The use of EGCG caused a number of adverse effects, including a decrease in the mitochondrial membrane potential of cells, decrease in GSH levels or increased ROS production which may induce cells apoptosis^9,21–23^.

The presence of heavy metals (HM) in the environment is a subject of research that is very often carried out by the scientific community these days. There has been a growing concern about it because of its increasing concentration, and therefore increasing danger to the health and life of living organisms, including humans. HMs are characterized by high toxicity, the ability to plant bioaccumulation and a long elimination half-life. The main sources of the increasing concentration of HMs in the environment are those connected with human activities, such as mining, transport, fossil fuel combustions, oil extraction, use of fertilizers, and production. Many studies have shown that metals such as lead, mercury, zinc, cadmium, arsenate, or vanadium are associated with ROS production leading to lipid peroxidation and alteration of antioxidant enzymes^24,25^.

**Figure 3:**
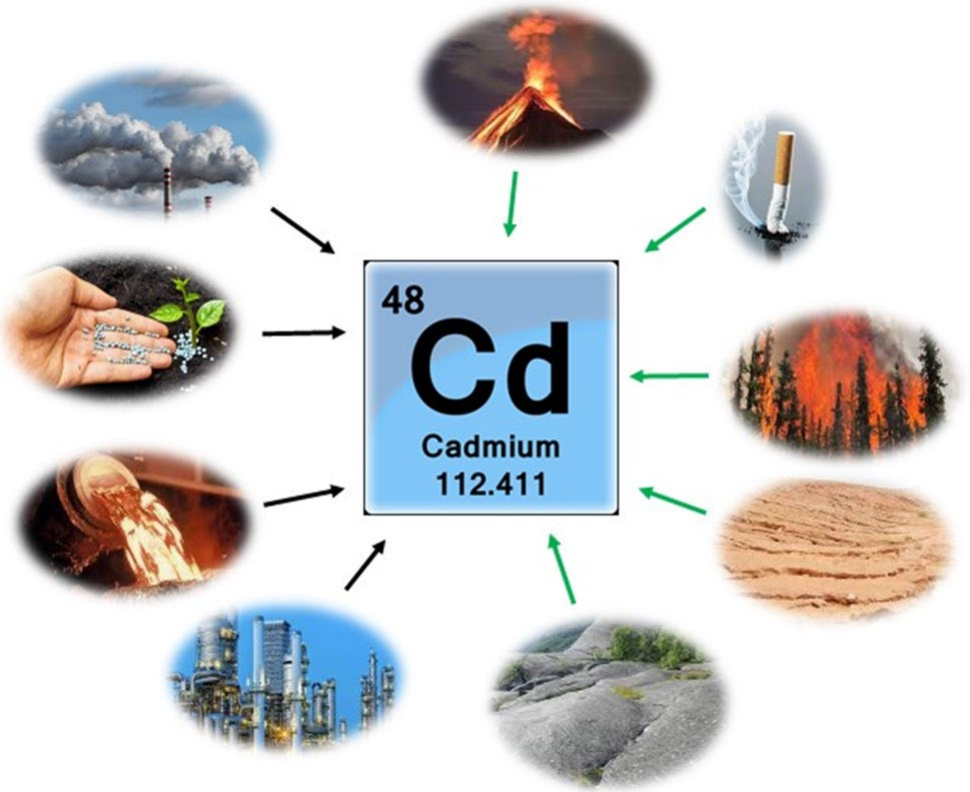
Natural (green arrow) and anthropogenic (black arrow) sources of Cd in the environment.

Cadmium (Cd) is one of the most dangerous HMs, according to the Agency for Toxic Substances and Disease Registry (ATSDR). Its emission to the environment is connected with natural and anthropogenic sources (Fig. 3). Emission related to human activity is mainly the combustion of fossil fuels, the use of phosphate fertilizers or copper and nickel smelting and refining. On the other hand, natural emission is caused by volcanic activity, forest fires and errosion and abrasion of rocks and soil^26^. Moreover, food (e.g. crab) is often reported as a source of human exposure to Cd because of its selective absorption by certain edible foods. Cd also contributes to human exposure through its presence in tobacco smoke^27^.

It has a toxic biological effect even at a concentration lower than that of any other metal. It should be noted that Cd can alter the cell cycle and promote tumorigenicity or apoptosis, depending on the exposure conditions^28,29^. The main molecular basis for Cd cytotoxicity is thought to be oxidative stress. It has been shown that cadmium can affect the activity of antioxidant enzymes—catalase (CAT), superoxide dismutase (SOD), glutathione reductase (GR)^30^. Most of the data suggest that oxidative stress is involved in developing damage to the liver^31,32^, kidneys^33,34^ and reproductive organs^35,36^. What should be noted, ovaries are the major target of the Cd accumulation, due to increased absorption of Cd by the gastrointestinal tract because of Fe deficiency, which often occurs during the female reproductive age^37,38^. The inhibition of oocytes development and ovary function may also be observed^39^. Moreover, the inhibitions of sex steroids production and necrosis of ovarian may also occur^40^. Furthermore, cadmium-induced oxidative stress has been implicated in cardiovascular system damage^41^, central nervous system damage^34^ and bones damage^42^ as well as eye and hearing damage (blindness and loss of hearing)^43–45^. Furthermore, Cd-induced changes in DNA methylation were shown that can contribute to some disorders, such as tumors^46,47^.

The Chinese hamster ovary (CHO-K1) cell line is commonly used in toxicological studies, e.g. to assess the toxicity of various environmental agents, such as the heavy metals, nanoparticles or mycotoxins^48–51^. It is an immortalized but non-cancerous cell line characterized by a high proliferation efficiency and the short doubling time^51,52^. The CHO-K1 line has been repeatedly used in in vitro studies, e.g. to assess the cytotoxicity of organic transition metal complexes^53^, to evaluate the antioxidant capacity of cells treated with heavy metals^54^ or in screening for cytotoxicity of inorganic heavy metal compounds^54,55^.

Available literature is unclear on EGCG's effect on heavy metal toxicity, especially in in vitro studies. Therefore, the aim of the present study was to investigate the effect of EGCG applied at physiologically achievable concentrations^56–58^ on the prooxidant and apoptotic effects of Cd in the CHO-K1 cell model. For this reason, the following analyzes were performed: (a) resazurin assay for cells viability determination, (b) CellTiter-Glo® Luminescent Cell Viability Assay for ATP content determination (c) Intracellular Total ROS Activity Assay for assessing the overall level of intracellular ROS activity, (d) MitoPT TMRM Assay to evaluate the mitochondrial membrane potential and (e) Annexin V-FITC Apoptosis Assay apoptosis/necrosis process assessment.

## Materials and methods

### Reagents

Dulbecco’s Modified Eagle Medium/Nutrient Mixture F-12 (DMEM/F-12) (cat. No. 31330095) was purchased from ThermoFisher Scientific (Waltham, Massachusetts, USA), fetal bovine serum (FBS, cat. No. F9665), antibiotic antimycotin solution (100×) (cat. No. A5955), trypsin–EDTA solution (0.05%) (cat. No. T3924), cadmium chloride (CdCl_2_, cat. No. 202908), (−)-epigallocatechin gallate (EGCG, cat. No. E4143) and In Vitro Toxicology Assay Kit, Resazurin Based (cat. No. TOX8-1KT) were purchased from Sigma-Aldrich (St. Louis, Missouri, USA). Intracellular Total ROS Activity Assay Kit (cat. No. 9144), Hydrogen Peroxide Fluorescent Detection Kit (cat. No. 9131), MitoPT® TMRM Mitochondrial Depolarization Assay Kit (cat. No. 9105) and the Annexin V-FITC Apoptosis Detection Kit (cat. No. 9124) were purchased from Immunochemistry Technologies, (Bloomington, MN, USA).

### Cell culture

The CHO-K1 cell line (cat. No. 85051005) was purchased from Sigma-Aldrich (St. Louis, Missouri, USA). The cells were cultured in a humidified incubator (CO_2_ incubator HERAcell 150i, Thermo Scientific, Germany) at 37 °C and 5% CO_2_, in DMEM/F12 with 5% FBS, 100 U/mL penicillin, 100 mg/mL streptomycin, and 0.25 µg/mL amphotericin B. Cultures were passaged using 0.05% trypsin solution. The observations were carried out with the use of a phase-contrast microscope (Olympus, model IX73, Japan). All procedures that require sterile conditions were carried out in a laminar flow cabinet (Herasafe laminar flow cabinet, model KS, Thermo Scientific, Germany).

### Cd and EGCG concentrations

Two concentrations of each reactant were used for the analyses. For CdCl_2_, these were concentrations of 5 and 10 µM, while for EGCG, concentrations of 0.5 and 1 µM were used. The choice of concentrations of both Cd and EGCG was preceded by preliminary analyses of the cellular response to different concentrations of these agents (Supplementary Fig. S1). In the case of Cd at concentrations of 5 and 10 µM, the effect of this metal on reduced cell viability after 24 h of exposure could be observed. The 20 µM concentration led to necrosis of the majority of cells in the population. Similar responses were noticed for EGCG- at a concentration of 5 µM where, a significant decrease in cell viability was also observed.

### Determination of cells viability

Resazurin is a non-toxic and stable redox dye in culture medium that is used by researchers to investigate cytotoxicity. The assay is based on the reduction of blue and nonfluorescent resazurin to pink and highly fluorescent resorufin by mitochondrial oxidoreductases produced by living cells^59^.

CHO-K1 cells were seeded at 1 × 10^4^ cells/well into 96-well plates containing DMEM/F-12 with 5% FBS and grown for 24 h in 37 °C in 5% CO_2_. In the next step, the old medium was replaced with the fresh one with the addition of EGCG (0.5 and 1 µM) and CdCl_2_ (5 and 10 µM), in combination or separately. After the next 24 h, the well medium was changed for the fresh DMEM/F-12 without the FBS and with 10 µL of resazurin, and incubated for 3 h. The absorbance was read by microplate reader (Synergy 2, BioTek Instruments Inc., Winooski, Vermont, USA) at 600 nm (690 background). Data from the resazurin assay are expressed as a percentage of control cells. They were calculated as follows Atest/Acontrol × 100% (Atest: absorbance of cells treated with Cd or EGCG, Acontrol: absorbance of control cells). Cytotoxicity (cell injury) was indicated as an increase in percentages compared to control cells.

### Determination of ATP content

The amount of ATP presented was determined with the CellTiter-Glo® Luminescent Cell Viability Assay (cat. No. 7570, Whitehead Scientific, Promega). The assay determine the number of metabolically active cells on quantitation of the ATP. It is based on the luminescent signal emitted by the thermostable luciferase, which is proportional to the amount of active cells and amount of ATP.

For the assay, CHO-K1 cells were seeded at 1 × 10^4^ cells/well into 96-well black plates containing DMEM/F-12 with 5% FBS. Cells grown for 24 h at 37 °C in 5% CO_2_. After that, the medium was replaced with the fresh medium containing EGCG (0.5 and 1 µM) and CdCl_2_ (5 and 10 µM), in combination or separately. The next day, the CellTiter-Glo® Luminescent Cell Viability Assay was performed according to the manufacturer protocol. The luminescence signal was measured in microplate reader (Synergy 2, BioTek Instruments Inc., Winooski, Vermont, USA).

### Changes in intracellular ROS levels

Total ROS activity was determined with the Intracellular Total ROS Activity Assay Kit (cat. No.9144, ImmunoChemistry Technologies, Bloomington, MN, USA). In the reaction, the key reagent, Total ROS Green, is oxidized from non-fluorescent form to a molecule with fluorescent properties, in the presence of ROS produced by the cells.

For the assay, 1 × 10^4^ cells per well in DMEM/F-12 with 5% FBS were seeded into 96-well black plates and cultured for 24 h at 37 °C in 5% CO_2_. Next, the medium was removed and cells were incubated for 30 min in ROS Green in a 1:50 v/v ratio. After that, the cells were washed with pure DMEM/F-12. The cells were then treated with Cd (5 and 10 µM), alone or together with EGCG (0.5 and 1 µM), for 1 h at 37 °C in 5% CO_2_ in the dark. The fluorescence signal was measured in a microplate reader (Synergy 2, BioTek Instruments, Inc. USA) using excitation and emission wavelengths of 485 and 528 nm, respectively. The measurements were made after 1 h, 2 h and 3 h of incubation with the fluorescence dye. Data (ROS levels) were expressed as a percentage of control cells.

### Assessment of mitochondrial membrane potential

For mitochondrial membrane potential measurements the MitoPT TMRM Assay (cat. No. 9105, ImmunoChemistry Technologies, Bloomington, MN, USA) was used, with TMRM (tetramethylrhodamine methyl ester) as a fluorescent dye. In healthy cells, TMRM accumulates in active mitochondria with intact membrane potential and emits red–orange fluorescence. In apoptotic damaged cells, mitochondria lose their membrane potential, and TMRM accumulations in mitochondria decrease which is associated with the disappearance of fluorescence emission. As a result, healthy cells would generate fluorescence unit outputs of orange fluorescence higher than those of the apoptopic ones.

For the assay, cells (1 × 10^4^ per well) were seeded into 96-well black plates in DMEM/F-12 containing 5% FBS and cultured for 24 h at 37 °C in 5% CO_2_. Subsequently, cells were treated with Cd, alone or in combination with EGCG in the same concentrations as in the previous tests, for 24 h at 37 °C in a 5% CO_2_. The supernatant was then replaced with a 200 nM MitoPT working solution, and the cells were incubated for 30 min at 37 °C in the dark. Cells were washed with Assays Buffer and then placed in a new portion of Assay Buffer until analysis. The fluorescence signal was measured in a microplate reader (Synergy 2, BioTek Instruments, Inc. USA) after 1 h and 24 h, using excitation and emission wavelengths of 530 nm and 590 nm, respectively.

### Detection of cell apoptosis

Apoptosis was assessed using flow cytometry (Annexin V-FITC Apoptosis Assay). CHO-K1 cells were seeded in 25 cm^2^ culture flasks and incubated at 37 °C in 5% CO_2_ for 48 h until they had reached 90% confluence. Next, the cells were treated for 24 h with Cd, alone or in combination with EGCG (as previously). After that cells were pelleted by centrifugation (200×*g* for 10 min) in PBS and resuspended in ice cold binding buffer. As a positive control, cells incubated with 3% formaldehyde in culture medium on ice for 30 min were used. Then cells were incubated with Annexin/Propidium Iodine for 10 min in the dark and analyzed by flow cytometry (BD FACSCalibur). The experiment was performed in three independent replicates.

### Caspase-3 and -9 activity assay

Caspases activities in cells were determined with the commercially available Caspase-Glo 3/7 Assay and Caspase-Glo 9 Assay (cat. No. G8090 and G8210, Whitehead Scientific, Promega). Assays provides luminogenic reagent which generate the luminescent signal produced by luciferase. The emitted signal is proportional to both caspases signal, which play a key role in the apoptopic pathway of mammalian cells.

For the assay, 1 × 10^4^ cells per well in DMEM/F-12 with 5% FBS were seeded into 96-well black plates and cultured for 24 h at 37 °C in 5% CO_2_. The next day, cells were treated for 3 h at 37 °C in 5% CO_2_ in the dark, with Cd and EGCG in the same combinations as in apoptopic assay. In the next step, the Caspase-Glo 3/7 Assay and Caspase-Glo-9 Assay were performed according to the manufacturer protocol. The luminescence signals were measured after 30 min of incubation, in microplate reader (Synergy 2, BioTek Instruments Inc.). Analysis were made in three independent repetitions.

### Statistical analysis

Data were analyzed using the Statistical Package for the Social Sciences (IBM Corp. Released 2020. IBM SPSS Statistics for Windows, Version 27.0. Armonk, NY: IBM Corp). First, all data were checked for outliers with Tuckey fence. A Mann–Whitney U (M–W) test was performed to detect the effects of Cd, EGCG, and combination of Cd and EGCG on the measured parameters.

## Results

### EGCG adds to the cytotoxicity of Cd

**Figure 4:**
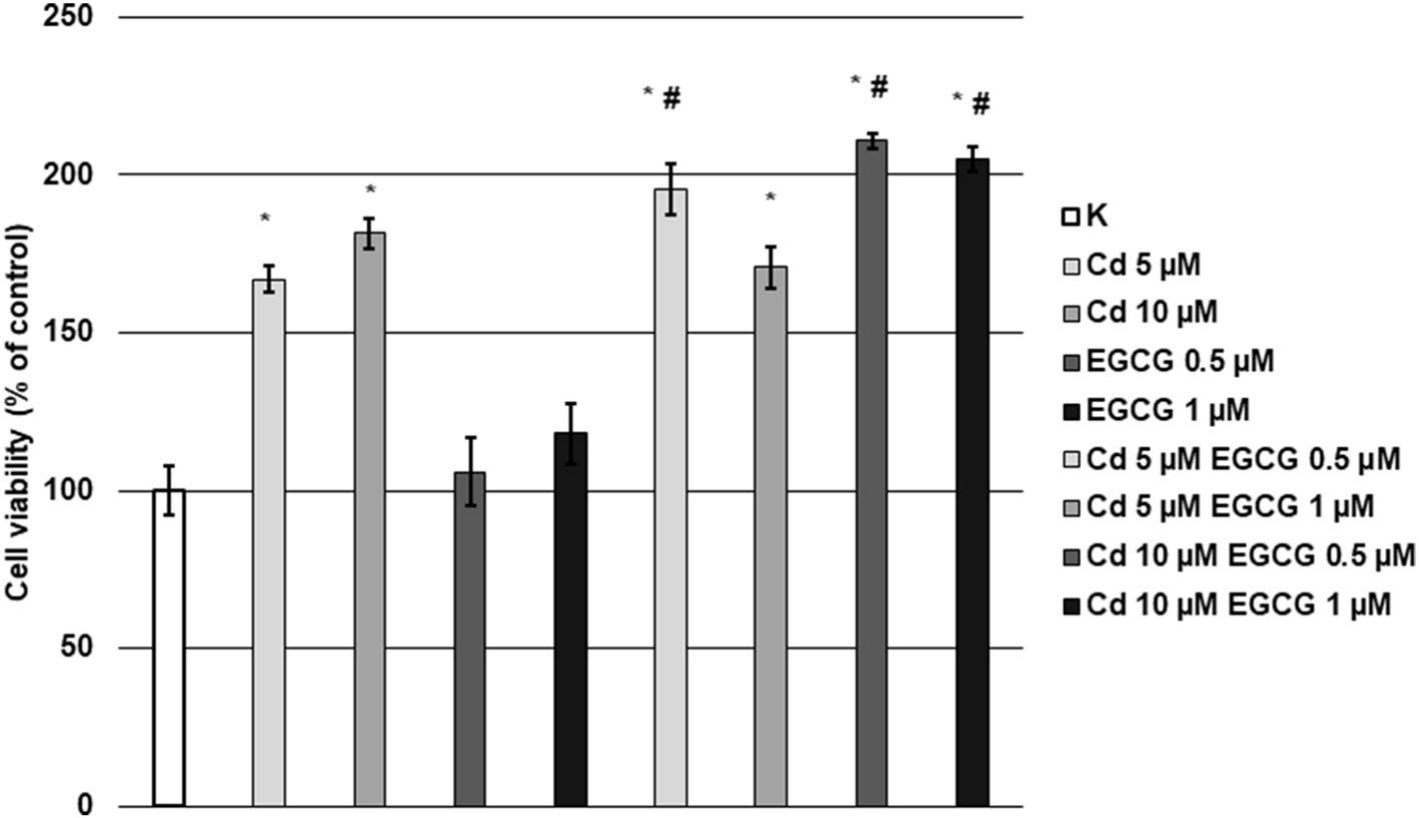
Effect of Cd, EGCG and Cd + EGCG on CHO-K1 cells viability, measured with resazurin assay. The absorbance of resazurin in control cells was taken as 100%. Results are presented as a percentage of control cells and represented as mean + SD derived from three independent experiments. Cytotoxicity is indicated by an increase in percentage values, compared to the control cells; *Significantly higher than the control; ^#^Significantly higher than Cd alone (Mann–Whitney U test, *p* < 0.05).

The resazurin assay was used to evaluate the cytotoxic effect of Cd, EGCG, and the interaction of both substances in CHO-K1 cells. In cells treated with CdCl_2_ (5, 10 µM) or EGCG (0.5, 1 µM) alone, a significant increase (*p* < 0.05) in absorbance values, compared to control, was observed. Further, cotreatment of cells with 5 µM CdCl_2_–0.5 µM EGCG, 10 µM CdCl_2_–0.5 µM EGCG and 10 µM CdCl_2_–1 µM EGCG showed a significant increase (*p* < 0.05 M–W test) in cytotoxicity, compared to respective Cd treatments without EGCG addition. (Fig. 4).

### Effect of EGCG and Cd cotreatment on ATP amount

**Figure 5:**
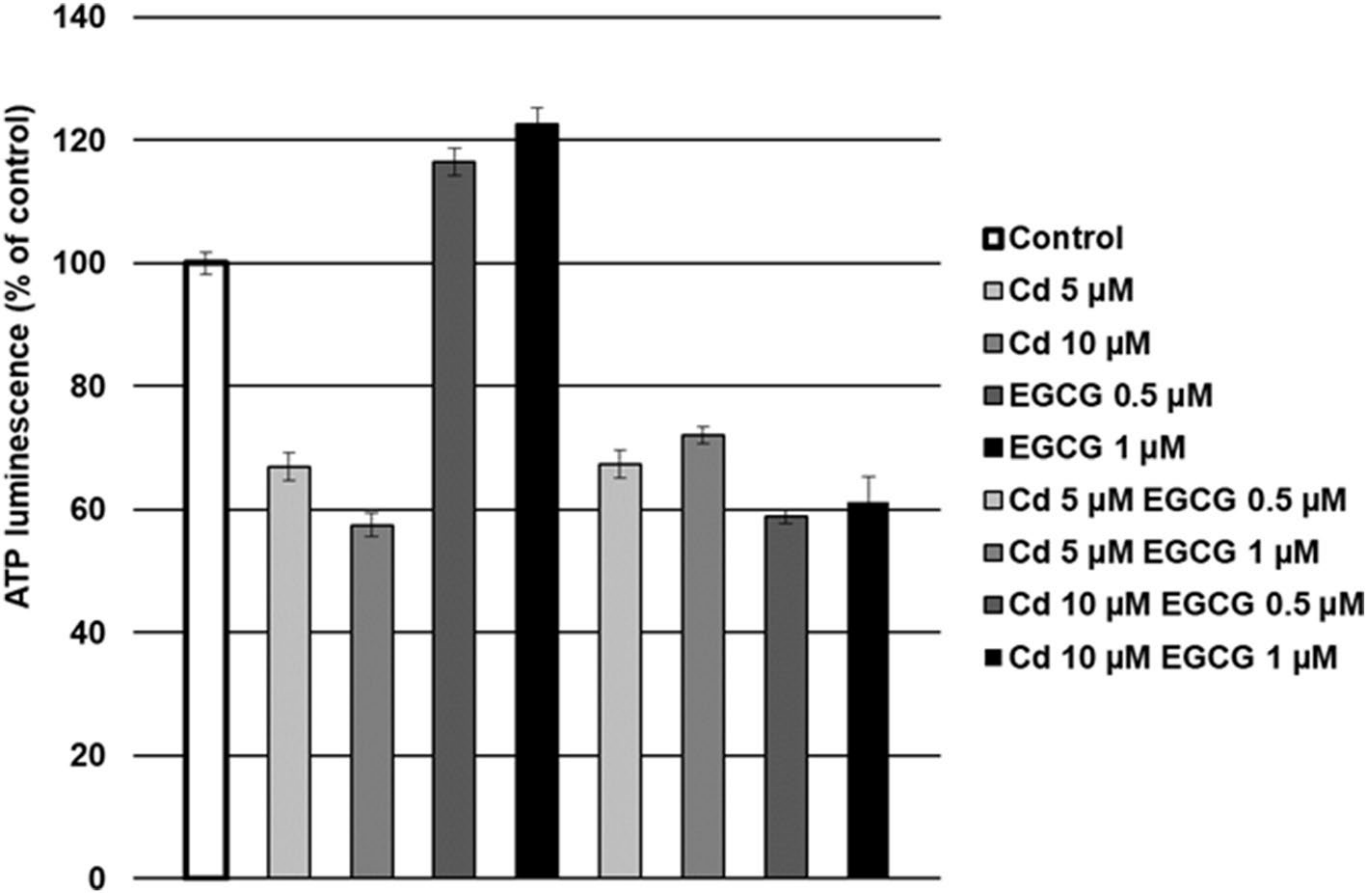
Effect of Cd, EGCG and Cd + EGCG on ATP production by CHO-K1 cells, measured with luciferase reaction. The luminescence of control cells was taken as 100%. Results are presented as a percentage of control cells – the higher value, the higher amount of ATP was detected. Results represented as mean + SD derived from three independent experiments. *Significantly higher than the control; ^#^Significantly higher than Cd alone (Mann–Whitney U test, *p* < 0.05); lack of significance in the presented results.

Cells treated with CdCl_2_ (5, 10 µM) was characterized with the decreased in ATP content, compared to the control samples. The lack of the protective effect of EGCG on cells treated with EGCG–Cd simultaneously was observed. Only in the case of 5 µM CdCl_2_–1 µM EGCG variant, the insignificant slight increase in the luminescence value was observed. In the remaining variants, the results of the cotreatment remained at a similar level as for the action of Cd alone (Fig. 5).

### Cd and EGCG cotreatment influences ROS production in CHO-K1 cells

**Figure 6:**
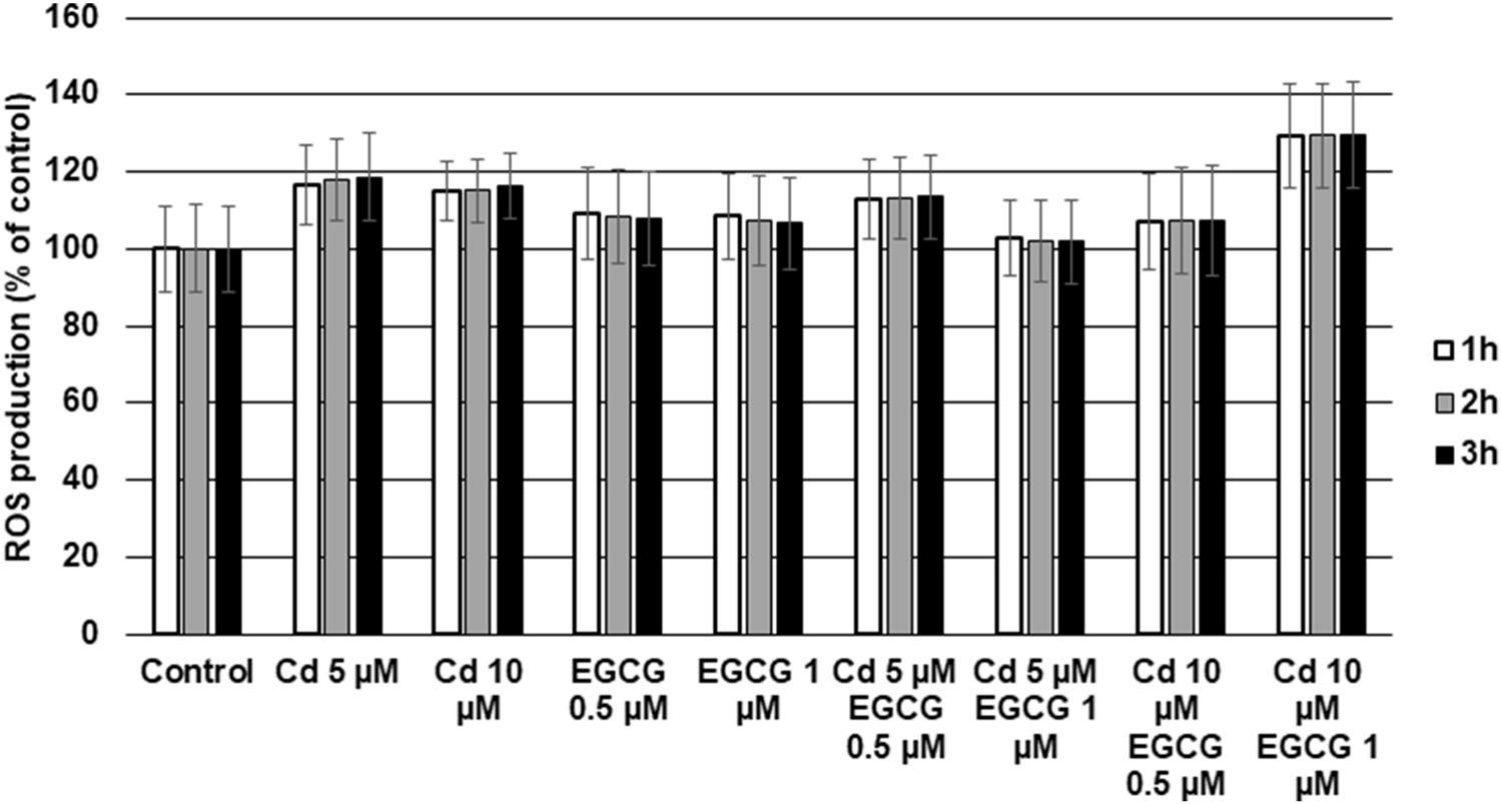
Effect of Cd, EGCG and Cd + EGCG on ROS production. The fluorescence of the Total ROS Green dye in control cells was taken as 100%. Results were expressed as a percentage of control cells- the higher value, the higher the content of ROS in cells. They represented as mean + SD derived from three independent experiments. *Significantly higher than the control; ^#^significantly higher than Cd alone (Mann–Whitney U test, p < 0.05); lack of significance in the presented results.

Figure 6 present the results of ROS production by Cd-treated CHO-K1 cells in the presence and absence of EGCG after 1 h, 2 h and 3 h of using Total ROS Green. Neither Cd (5 and 10 µM) nor EGCG (0.5 and 1 µM) had any significant effect on ROS generation compared to control cells. In turn, among all variants with cells coincubated with Cd and EGCG, an increase in ROS level was detected only in cells simultaneously treated with 10 µM CdCl_2_ and 1 µM EGCG compared to control cells. Coincubation with the remaining Cd and EGCG concentration variants (5 µM Cd–0.5 µM EGCG, 5 µM Cd–1 µM EGCG, 10 µM Cd–0.5 µM EGCG) had no significant effect on ROS generation compared to control.

### Effect of EGCG mitochondrial membrane potential (MMP) in CHO-K1 cells

**Figure 7:**
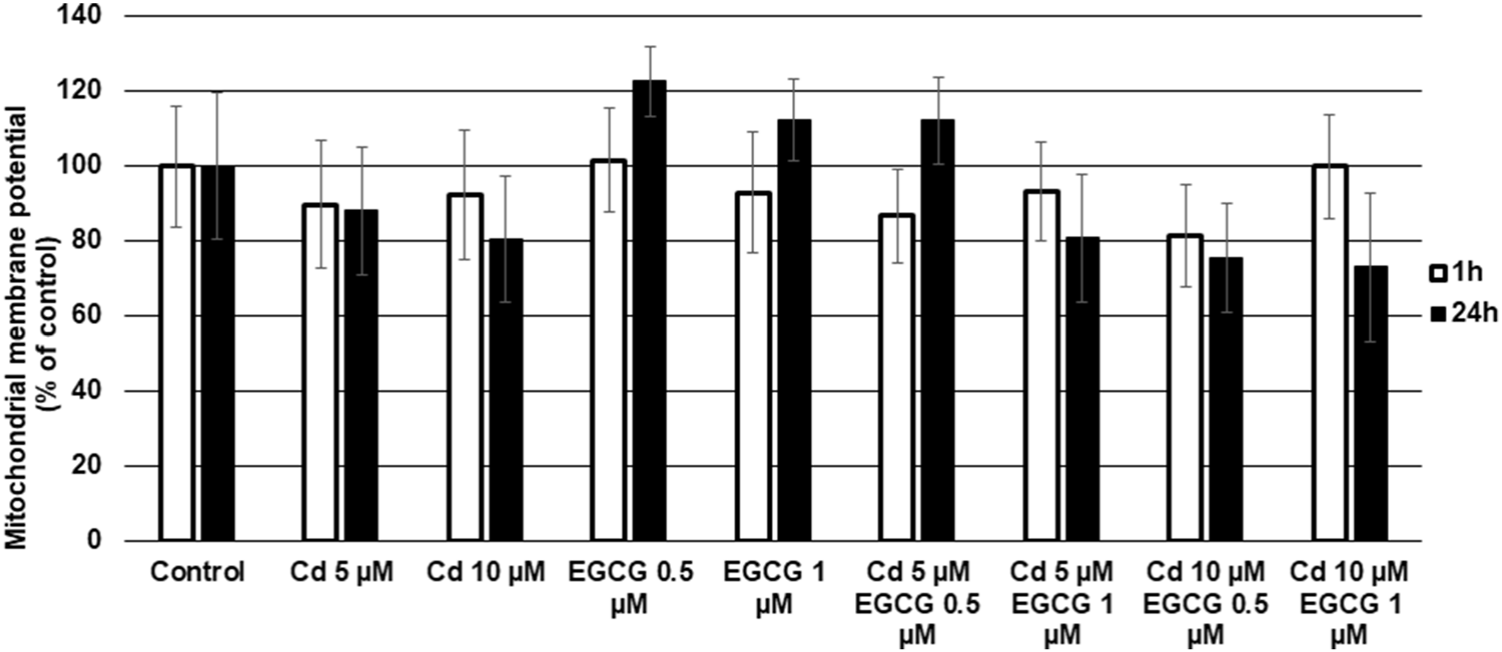
Effect of Cd, EGCG and Cd + EGCG on mitochondrial membrane potential. The fluorescence of the TMRM dye in control cells was taken as 100%. Results were expressed as a percentage of control cells- the higher value, the lower the content of apoptopic cells. Results represented as mean + SD derived from three independent experiments; *Significantly higher than the control; ^#^Significantly higher than Cd alone (Mann–Whitney U test, *p* < 0.05); lack of significance in the presented results.

The results after 1 and 24 h of exposure of CHO-K1 cells to CdCl_2_ at tested concentrations with and without EGCG are presented in Fig. 7. Exposing cells to Cd (5 or 10 µM) or EGCG (0.5 or 1 µM) alone resulted in a slight decrease or slight increase in MMPs, respectively, especially after 24 h of exposure to the tested compounds. However, these differences were not statistically significant when compared to the control. Similarly, co-incubating cells with Cd and EGCG did not induce significant changes in MMPs compared to control cells or the corresponding controls for Cd 5 µM and Cd 10 µM.

### Apoptopic/necrotic effect of EGCG on Cd-treated cells

**Figure 8:**
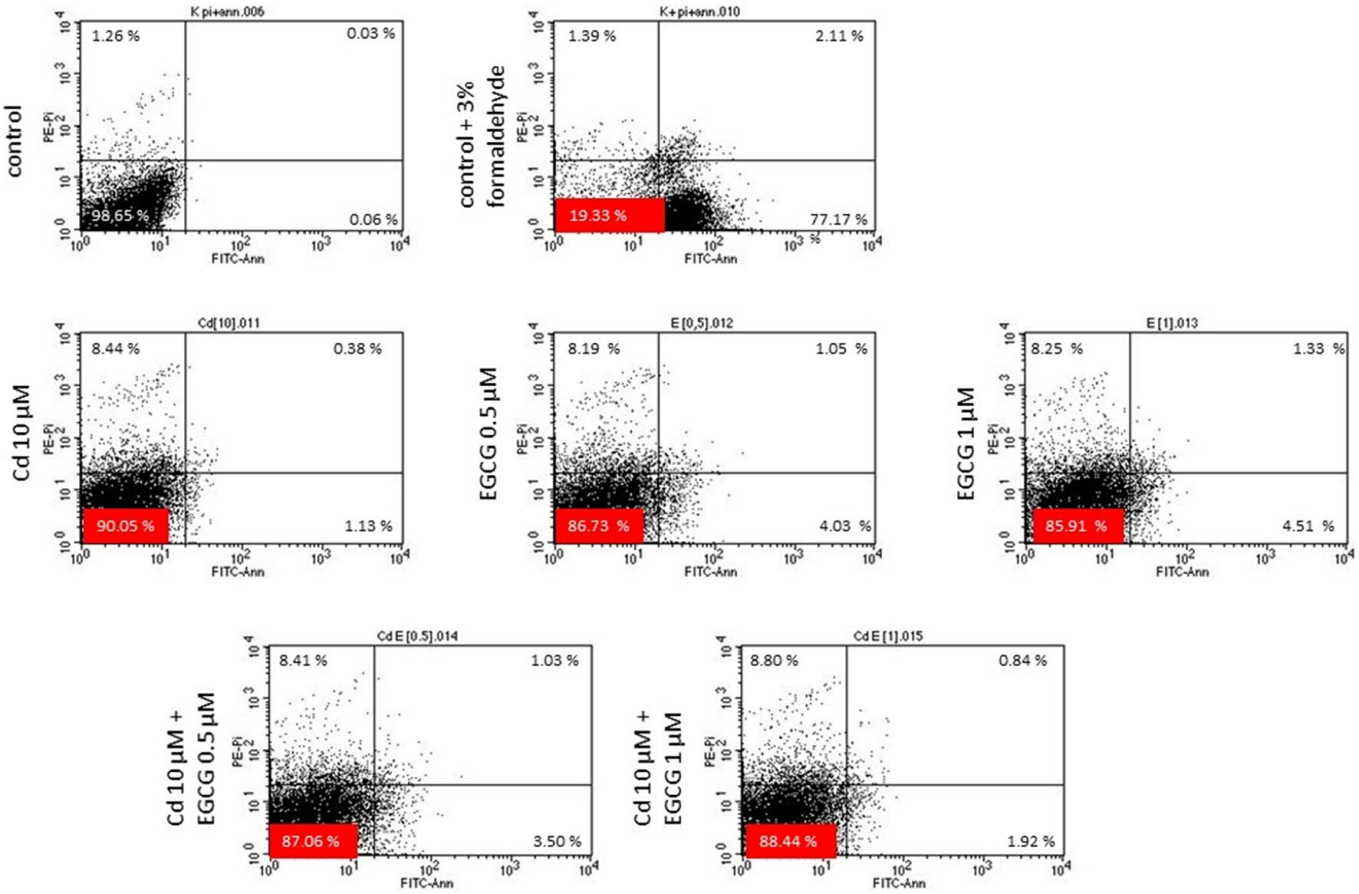
Apoptosis-inducing effect of CHO-K1 cells treated with Cd, EGCG and Cd + EGCG detected by Annexin V/PI double staining. Apoptosis was quantified using flow cytometry after staining with annexin V/PI. Representative scatter plots of PI (y-axis) versus annexin V (x-axis).

To determine the potential protective effect of EGCG on Cd-treated cells, flow cytometry analyses were performed using the annexin V- and PI-staining (Fig. 8, Supplementary Fig. S2). Figure 8 shows results representative of three similar experiments. Early apoptosis was observed in 0.03% of the cells in the control and 82.19% in the positive control. Cells treated with only 0.5 and 1 µM EGCG and 10 µM Cd showed 1.35%, 1.85% and 0.63%, cells respectively, in early apoptosis. Furthermore, an increase in the amount of early apoptotic cells (compared to the control with 10 µM Cd) was observed in the variants with coincubation. The observed value of 1.19% in the 10 µM Cd–0.5 µM EGCG variant was higher than in the 10 µM Cd–1 µM EGCG variant (0.96%). The analysis showed that in cells treated with 10 µM Cd^2+^ the amount of necrotic cells was higher than the in control variant, 4.35% in cells with Cd^2+^ and 1.08% in control and 0.71% in positive control. EGCG at 0.5 and 1 µM also induced necrosis at 3.22 and 3.25%, respectively. Further, the simultaneous effect of Cd and EGCG on cells led to an increase in the presence of necrotic cells, in the variant Cd^2+^ 10 µM–EGCG 1 µM variant, with the value 4.45%.

### Cells cotreatment with Cd/EGCG influenced the Caspase-3 and Caspase-9 acitivity

**Figure 9:**
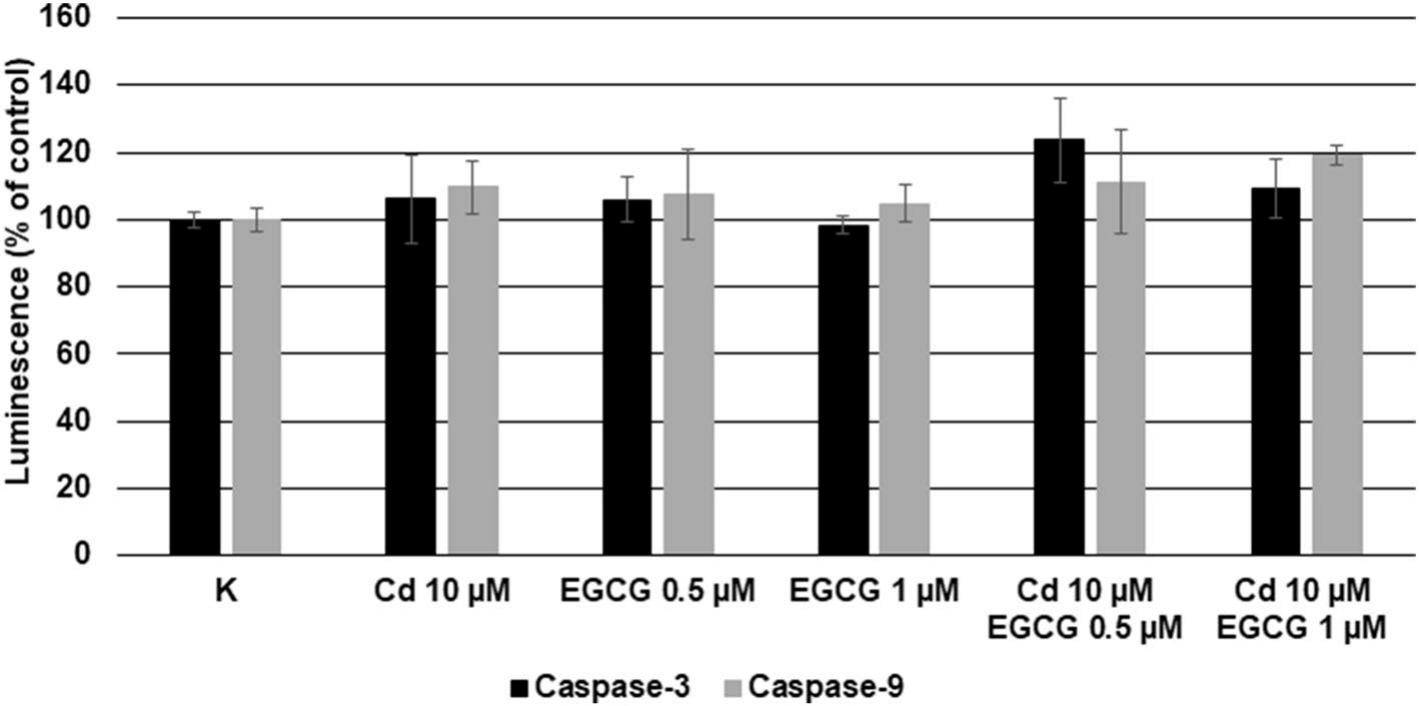
Effect of Cd, EGCG and Cd + EGCG on Caspase-3 and Caspase-9 activity. The luminescence of control cells was taken as 100%. Results are presented as a percentage of control cells – the higher value, the higher Caspase-3 (black colour) and Caspase-9 (grey colour) activity were. Results represented as mean + SD derived from three independent experiments. *Significantly higher than the control; ^#^Significantly higher than Cd alone (Mann–Whitney U test, *p* < 0.05); lack of significance in the presented results.

Figure 9 present the Caspase-3 and Caspase-9 activity in cells treated with Cd (10uM) and EGCG (0.5 and 1 uM), together or alone. As presented, cells treatment with Cd and EGCG separately resulted only in slight increase in the Caspase-3 and -9 activities, compared with control cells. Moreover, there were no protective effect of EGCG on cells treated with EGCG–Cd simultaneously. In the case of Caspase-3, the highest luminescence was observed in variant Cd 10-EGCG 0.5, the increase in EGCG concentration resulted in the reduction of Caspase-3 acitivity. On the contrary, the increase of Caspase-9 activity was observed together with increasing EGCG concentration. However, no statistical significance was observed in any of the variants. The results obtained are in line with the trend observed in apoptosis assay.

## Discussion

In our study the effect of EGCG against Cd-induced toxicity of CHO-K1 cells was tested. Cells were treated with Cd (5 and 10 µM), EGCG (0.5 and 1 µM), and Cd–EGCG together at the same time. It should be noted that the low levels of EGCG in the research correspond to concentrations that can be obtained in the body from a standard diet^56–58,60^. This could give a better answer to its effect on cells, especially for oral ingestion.

The doses of EGCG used in the research increased the toxicity of Cd under our experimental conditions. This was evidenced by increased cytotoxicity (in all variants tested), decreased amount of generated ATP by cells, increased ROS production (10 µM Cd–1 µM EGCG variant) and slight disturbances in MMP. Moreover, an increased number of early and late-apoptotic cells was observed in cultures treated simultaneously with Cd and EGCG compared to cells treated with Cd alone.

In the available literature, there can be found results of cytotoxicity studies presenting a similar trend to that observed in our study. Bondad and Kurasaki^8^ showed the enhanced toxicity of Cd in the presence of EGCG in PC12 cells (cells derived from transplantable rat pheochromocytoma). They observed that EGCG at dose 1.5 µM with 5 µM Cd caused increased LDH activity and decreased cell viability (in test with trypan blue) suggesting cell membrane damage, as compared to cells treated with Cd alone. Such results were explained by the chemical structure of EGCG and its behavior instability and ability to ROS generation under certain conditions. Factors such as pH (considered a critical factor), the presence of metal ions, temperature, and oxygen content all negatively affect the stability of EGCG, which can lead to greater ROS production. For example the increased production of H_2_O_2_ in the culture medium in the presence of EGCG may result in an increased toxic effect of the Cd–EGCG cotreatment^61–63^.

Stronger toxic effect of Cd in doses 10, 30 and 50 µM on cells in the presence of 50 µM EGCG was also observed by Yu et al.^64^, where the viability of human prostate cancer cells (PC-3) (measured by the MTT assay) was significantly reduced when both factors were added, compared with cells where Cd was added alone. Analogous results were obtained by Zhang et al.^65^, who also carried out his analyses on PC-3 cells. In addition to the enhanced toxicity of Cd (5, 10, 20, 40 µM) in the presence of EGCG (80 µM), which increased with increasing concentrations of both Cd and EGCG, there was a reduction in cell viability under 80 µM of EGCG added alone (measured by the MTT assay) of approximately 40%. In addition, Yu et al.^64^ presented different cells responses to the order of addition of both components. In the variant that was also used in our experiment (EGCG and Cd were added to the cells at the same time), a reduction in cell viability was observed, compared to cells treated with Cd alone. This agrees with the results obtained in our research. Such reactions of Cd + EGCG action have been explained by researchers in various ways. First, the reduction in cell viability as a result of EGCG + Cd interactions were due to the catechin-metal ion interaction leading to an imbalance in cell metabolism and thus inhibiting cell growth. Secondly, the direct reaction of the two compounds with each other led to an increase in Cd content in cells, leading to a decrease in cell numbers^64^. Third, the reduction in cells number may be due to blocking of the effectors involved in the mitochondrial apoptosis pathway by the interaction of Cd + EGCG^65^. Cd can induce cell apoptosis through the apoptotic pathway in which mitochondria are involved (activation of caspase-9 responsible for carrying out apoptosis in mitochondria). The authors observed a decrease in mitochondrial membrane potential (which was also observed in our study, in variants Cd 5 µM–EGCG 0.5 µM (1 h), Cd 5 µM–EGCG 1 µM (1 h and 24 h), Cd 10 µM–EGCG 0.5 µM (1 h and 24 h) and Cd 10 µM–EGCG 1 µM (24 h), Fig. 7), a decrease in ATP content (observed in all variants with cotreatment, Fig. 5) and the release of caspase-9 (Fig. 9) under the influence of Cd + EGCG, resulting in damage to the cells studied.

There are reports in the literature of negative effects of EGCG alone on cells, which was also observed in the results of our analyses. Sonoda et al.^66^ in their investigation observed the reduced viability of A549 cells when the EGCG concentration was > 25 µM, where the IC50 (50% inhibition of cell growth) was noted at EGCG concentration of 36 µM. In our experiment, the concentrations which induced a decrease in the cell viability i.e. 0.5 and 1 µM EGCG were much lower, which may be due to the difference in the cells used and their sensitivity. The negative effect of EGCG on cells was also observed by Yang et al.^67^. The authors observed a decreasing cell viability of H661 (human lung cancer), H441 (lung cancer) and HT-29 (colon cancer) cell lines (measured using trypan blue stain) after 48 h incubation in EGCG concentrations ranging from 30 to 100 µM. Moreover, an increase in apoptosis index of H661 cells was observed together with increasing EGCG concentration—the content of apoptotic cells in the control, in 30 µM and 100 µM EGCG was 1.8, 3.2 and 12.4%, respectively. In our results an increase in apoptotic cells content (early + late apoptotic cells) was also noted, together with increasing EGCG concentration (0.08% in control cells, 1.70% in EGCG 0.5 µM and 2.33% in EGCG 1 µM). Results were confirmed by Wang and Lei^68^ who observed the significant inhibition of cells proliferation and apoptosis induction together with increasing EGCG concentration and its duration of action on cells. Such results may be a consequence of the behaviour of EGCG caused by the cell culture medium components. Researchers have shown that EGCG in culture media is unstable and produces significant amounts of H_2_O_2_. These results were confirmed for several types of culture media: DMEM, RPMI 1640, Ham F-12 and McCoy 5A^63,69^ of which DMEM showed generation of the highest amounts of H_2_O_2_^70^. As the concentration of EGCG as well as its duration of action in the DMEM increased, the amount of H_2_O_2_ generated was greater. In such a situation, depending on the cell type, it is possible to observe, among other things, an induction of apoptosis, an increase in cytotoxicity, an inhibition of cell growth or proliferation^70^. It is also noteworthy that, in a study conducted by Akagawa et al.^61^, EGCG showed the production of the highest amounts of H_2_O_2_ of all the polyphenols tested: Gallic acid (GA), Chlorogenic acid (CGA), Caffeic acid (CA), Catechin (C), Epicatechin (EC), Epicatechin gallate (ECG), Epigallocatechin (EGC) and aforementioned EGCG.

## Conclusions

The main conclusion of the study is that EGCG concentrations used in the research resulted in enhancement in inhibition of cell viability, function and increased apoptosis. Moreover, an increase in the content of cells in apoptosis was observed under the influence of EGCG alone. The possible effect of such a reaction could be instability of EGCG under cell culture conditions influenced by the components of the cell culture medium. However, such conclusions require further in-depth studies on the interactions of EGCG and metal ions and their effects on cells according to the prevailing conditions.

### Supplementary Information

Supplementary Figure 1.

Supplementary Figure 2.

## Data Availability

The datasets used and/or analysed during the current study available from the corresponding author on reasonable request.
