# Supplementary figures and images for: The physiological levels of epigallocatechin gallate (EGCG) enhance the Cd-induced oxidative stress and apoptosis in CHO-K1 cells
Source: Sci Rep. 2024 Jun 13;14:13625. doi: 10.1038/s41598-024-64478-7 (PMC11176361; doi:10.1038/s41598-024-64478-7)

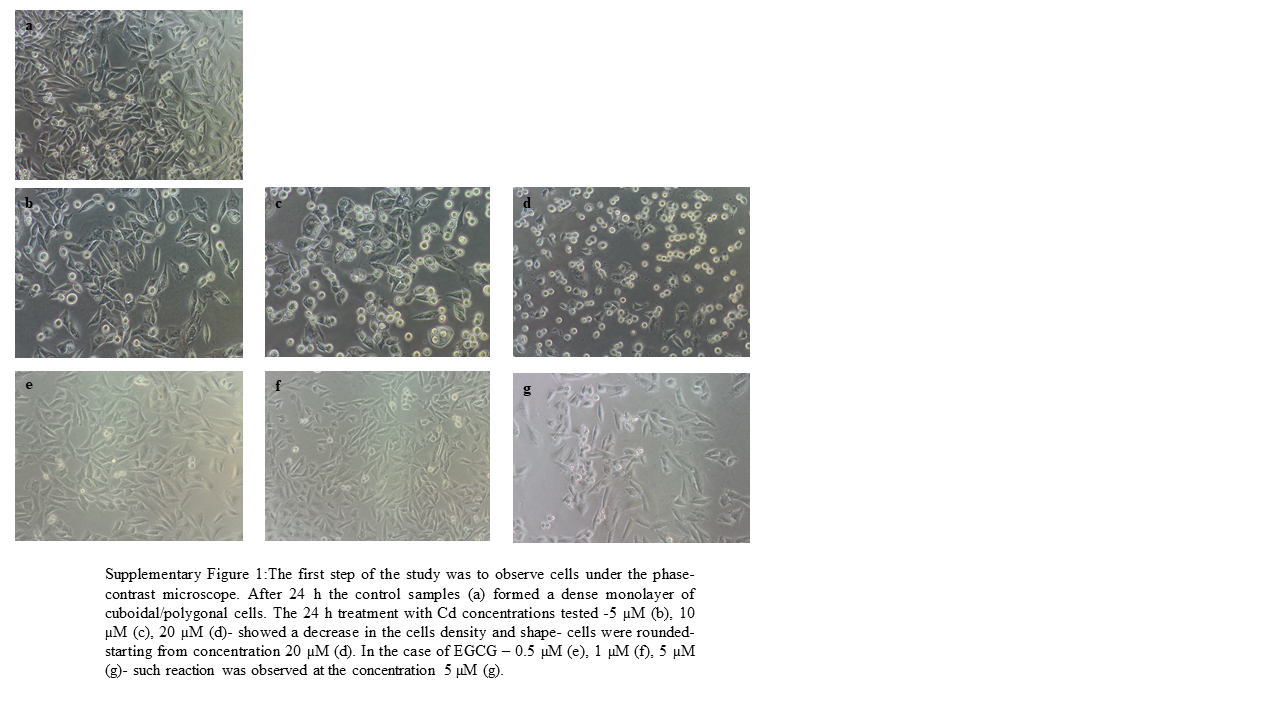

Supplement: Supplementary file 1 — Supplementary Figure 1. [file 41598_2024_64478_MOESM1_ESM.tif]

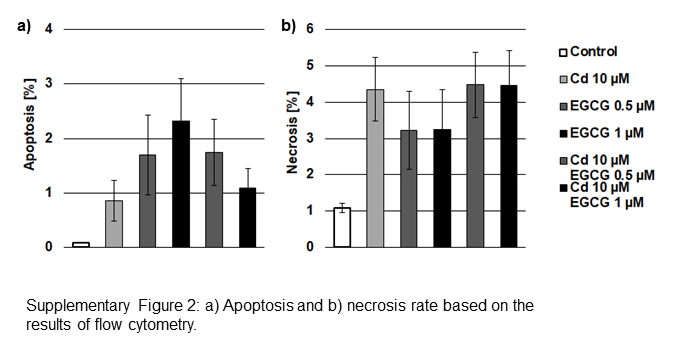

Supplement: Supplementary file 2 — Supplementary Figure 2. [file 41598_2024_64478_MOESM2_ESM.tif]
